# EHMTI-0042. 2003-2013. Trends in clinical characteristics and use of health care services of moh patients attending an headache centre in Italy

**DOI:** 10.1186/1129-2377-15-S1-D50

**Published:** 2014-09-18

**Authors:** P Rossi

**Affiliations:** 1 Headache Centre, INI, Grottaferrata (Rome), Italy

## Background and aim

Over the past 15 years clinical experience and scientific evidences about MOH has accumulated providing the basis for new simplified diagnostic criteria, treatment guidelines and possibly an increased awareness about this disorder. The aim of this study was to analyze the trends in prevalence, clinical severity and health care utilization in MOH patients attending a specialty headache centre.

## Methods

Data regarding the prevalence of MOH, the socio-demographic status (SES), the clinical characteristics (headache frequency, severity and related-disability, type of medication overused, duration of MOH, frequency of drug intake, complicated vs. simple form) and the health care use measures were extracted from the Headache clinic database.

## Results

The prevalence of MOH patients visited at the INI headache centre decreased over time (17.3%-5.7%, p<0.0001) due to a significant reduction in the proportion of MOH females (18%-6%, p<0.01. When compared with the 2013 MOH cohort, MOH patients visited in 2003 were significantly more disabled (MIDAS IV, 66 v 32%, p<0.001), had an higher frequency of headache (28.2 vs 24.3 days/month, p=0.03), had a higher drug intake (42 vs 26 drugs/month, p<0.01) had a longer duration of chronification (48 vs. 24 months), had a higher rate of complicated forms (73 vs 48%) and had used more frequently a preventive treatment (54% vs 32%, p=0.03).

## Conclusion

Over a ten-years period MOH has become less prevalent and the clinical profile has improved over time suggesting an earlier recognition of MOH and an improved access of these patients to specialists’ service.

No conflict of interest.

